# Supplementary material for: The genome sequence of the Provençal Short-tailed Blue, Cupido alcetas (Hoffmannsegg, 1804) (Lepidoptera: Lycaenidae)
Source: Wellcome Open Res. 2026 Jan 23;11:63. [Version 1] doi: 10.12688/wellcomeopenres.25791.1 (PMC12946697; doi:10.12688/wellcomeopenres.25791.1)
Supplement: Peer review history

# Reviewer report: Reviewer response for version 1

**DOI:** 10.21956/wellcomeopenres.28392.r146608

**Published:** 2026-02-27

**Authors:** Oliver Rupp

**Affiliations:** 1 Justus-Liebig-University, Giessengiessen, Germany

This article presents the diploid genome assembly, including the mitochondrial genome, of Cupido alcetas, a small butterfly. The sequencing methods, including PacBio HiFi and Hi-C sequencing, follow the state-of-the-art methods used for eukaryotic genome sequencing. The subsequent assembly pipeline included well established tools including hifiasm, for the initial assembly, YaHS for scaffolding, and MerquryFK and BUSCO for the quality assessment. The haplotype 1 was manually curated into a chromosome level assembly. All quality metrics point out a high quality assembly. The assembly pipelines are available and the data was submitted to the European Nucleotide Archive.

Are sufficient details of methods and materials provided to allow replication by others?

Yes

Is the rationale for creating the dataset(s) clearly described?

Yes

Are the datasets clearly presented in a useable and accessible format?

Yes

Are the protocols appropriate and is the work technically sound?

Yes

Reviewer Expertise:

eukaryotic genome assembly

I confirm that I have read this submission and believe that I have an appropriate level of expertise to confirm that it is of an acceptable scientific standard.

# Reviewer report: Reviewer response for version 1

**DOI:** 10.21956/wellcomeopenres.28392.r146614

**Published:** 2026-02-26

**Authors:** Olli-Pekka Smolander

**Affiliations:** 1 University of Helsinki, Helsinki, Finland

The manuscript describes the construction of a high quality chromosome level genome assembly for the Provencal Short-tailed Blue butterfly. The genome has been assembled based on PacBio HiFi and Hi-C data from a single female specimen yielding a primary assembly spanning 493.02 megabases. The assembly is organized into 26 chromosomal pseudomolecules including a unique sex chromosome arrangement involving the W and two Z chromosomes.

 The biological significance of the work is based on the conservation status of the species: it is threatened and near extinction in several European countries. The authors also highlight the taxonomic complexity and potential for misidentification with related species. However in the background-section text they mention that records from some regions have low reliability and likely represent a different expanding species without presenting evidence or citations for these claims. To enhance the scientific rationale the authors should clarify this. Is the morphological spot is sometimes insufficient for certain identification? This would allow more clearly justifying the need for these genomic data. Additionally, the mention of associations with the ant genera *Formica* and *Tapinoma* is a useful biological detail and the section would be even more cohesive if a brief sentence explained the significance of these interactions within the family.

 The methodology includes current benchmarks for reference quality genomes. The use of PacBio HiFi for sequencing and the Hi-C data for scaffolding is a high-level standard for such work. The assembly process and quality assessment has been done rigorously using appropriate tools.

 The manuscript provides sufficient experimental details such as the specific geolocation coordinates, collection date, laboratory procedures and software versions well documented. However, because this butterfly is very similar to other species the authors should mention the specific diagnostic criteria or tools used during sample acquisition to ensure the specimen was not an individual of the similar expanding species mentioned in the background. Including these details would strengthen the confidence in the taxonomic identification of the biological source material

 The data are well organized and adhere to open science standards. All raw data and the final assembly are deposited in the European Nucleotide Archive. Detailed assembly statistics are clearly tabulated and visualisations are provided.

 The article presents a technically robust genome assembly but would benefit from additional context and evidence in the text to be fully rigorous. Specifically the authors should provide citations or evidence for the claim of low reliability in existing records and the expansion of the related species. The methods should also clarify the identification process for the specific specimen used. Finally, the results describe a sex chromosome arrangement involving the W and two Z chromosomes. Adding a brief note of context regarding the nature or origin of the Z duplication would help readers appreciate the evolutionary significance of this chromosomal structure. Addressing these would further improve the manuscript and increase its value.

Are sufficient details of methods and materials provided to allow replication by others?

Yes

Is the rationale for creating the dataset(s) clearly described?

Yes

Are the datasets clearly presented in a useable and accessible format?

Yes

Are the protocols appropriate and is the work technically sound?

Yes

Reviewer Expertise:

Genomics, bioinformatics, ML/AI, computational biology, microbiome, gut-brain axis

I confirm that I have read this submission and believe that I have an appropriate level of expertise to confirm that it is of an acceptable scientific standard.

# Reviewer report: Reviewer response for version 1

**DOI:** 10.21956/wellcomeopenres.28392.r146615

**Published:** 2026-02-16

**Authors:** Melody Clark

**Affiliations:** 1 Natural Environment Research Council, Cambridge, UK

This Wellcome Open Research article  describes the haplotype-resolved genome of the short-tailed blue butterfly (female). The morphology distinguishing this specimen from closely related species is well described, as is the biology and biogeography. This species was sequenced under the European Project Psyche and is a priority for sequencing given its threatened and near extinction status in several European countries.

 All methods are well described with full acknowledgement of freely available methodologies. The PacBio HiFi and Hi-C assembly comprises 26 haplotype resolved pseudochromosomes including the W and Z1, Z2 sex chromosomes. The assembly includes contamination removal, in addition to some manual corrections.

 The description of the final assembly is well described using standard Open Source tools with the addition of chromosome painting of Meridian elements to illustrate chromosome evolution relative to the Lepidopteran ancestor. As with all Wellcome Sanger Institute produced genomes, this one is of a very high standard.

Are sufficient details of methods and materials provided to allow replication by others?

Yes

Is the rationale for creating the dataset(s) clearly described?

Yes

Are the datasets clearly presented in a useable and accessible format?

Yes

Are the protocols appropriate and is the work technically sound?

Yes

Reviewer Expertise:

transcriptomics and genomics of  invertebrates and fish

I confirm that I have read this submission and believe that I have an appropriate level of expertise to confirm that it is of an acceptable scientific standard.
